# Bridging Perception and Reasoning: An Evidence-Based Agentic System for Diagnosis and Treatment Recommendations of Vascular Anomalies

**DOI:** 10.3390/diagnostics16040621

**Published:** 2026-02-20

**Authors:** Yize Zhang, Yajing Qiu, Xiaoxi Lin

**Affiliations:** 1 Department of Plastic and Reconstructive Surgery, Shanghai Ninth People’s Hospital, Shanghai Jiao Tong University School of Medicine, Shanghai 200000, China; ezzh2407@gmail.com; 2 Shanghai Innovation Institute, Shanghai 200000, China

**Keywords:** deep learning, vascular anomalies, AI-assisted diagnostics, large language models, retrieval-augmented generation, agentic system, evidence-based reasoning

## Abstract

**Background:** Vascular anomalies (VAs), including hemangiomas and vascular malformations, present a significant diagnostic challenge due to their high prevalence, complex classification (nearly 100 subtypes), and visual mimicry. Current Multimodal Large Language Models (MLLMs) struggle in this specialized domain, often failing to capture fine-grained visual features or lacking evidence-based reasoning. To address these limitations, we introduce HevaDx, an agentic diagnostic system that explicitly decouples visual perception from clinical reasoning. **Methods:** Leveraging a newly constructed large-scale dataset of VA patients, HevaDx employs a lightweight visual specialist for precise feature extraction and a reasoning specialist equipped with Retrieval-Augmented Generation (RAG) for therapeutic planning. This cooperative architecture mitigates the “reasoning gap” observed in end-to-end models by grounding decisions in up-to-date clinical guidelines. **Results:** Experimental results demonstrate that HevaDx achieves high performance with a top-3 diagnostic accuracy of 94.8% and a treatment recommendation accuracy of 83.3%. **Conclusions:** By bridging visual precision with transparent, verifiable logic, HevaDx offers a reliable framework for AI-assisted management of vascular anomalies.

## 1. Introduction

Vascular anomalies (VAs), represented by hemangiomas and vascular malformations, include a broad spectrum of disorders that, despite a high prevalence of approximately 2.2% worldwide, present a significant diagnostic challenge [1,2,3]. These disorders encompass nearly 100 distinct subtypes with vastly different pathogenic mechanisms and clinical courses [4]. However, they often exhibit strikingly similar visual appearances. For example, it could be a severe challenge for clinicians to distinguish between a deep infantile hemangioma and a venous malformation, particularly in primary care and resource-limited settings. Misdiagnosis is critical, as therapeutic approaches differ markedly; a strategy effective for a tumor may be ineffective or harmful for a malformation [5,6,7,8]. Consequently, there is an urgent need for intelligent systems capable of assisting clinicians in both precise differentiation and evidence-based treatment planning.

Formally, the International Society for the Study of Vascular Anomalies (ISSVA) organizes this heterogeneous group into two biologically distinct lineages: vascular tumors, driven by endothelial hyperproliferation (e.g., infantile hemangioma), and vascular malformations, resulting from structural errors in morphogenesis [4]. The latter category is further stratified by the predominant vessel type, encompassing capillary malformations (CM)—represented by “port-wine stains” (PWSs)—as well as venous, lymphatic, and arteriovenous malformations. While this taxonomy provides a rigorous pathological framework, the visual boundaries between these entities are often blurred in clinical practice. The subtle morphological overlap between a proliferative tumor and a structural malformation poses a significant cognitive burden for general practitioners, necessitating intelligent systems capable of navigating this complex diagnostic landscape.

The recent surge in Multimodal Large Language Models (MLLMs/LLMs) [9,10,11,12] has sparked hope for automated “generalist” medical assistants [13,14]. Ideally, such models would ingest lesion images and patient history to output comprehensive clinical decisions. However, current state-of-the-art (SOTA) open-source models struggle in this specialized domain. Our preliminary investigations reveal that generic MLLMs fail to capture the subtle, fine-grained visual features required to distinguish VA subtypes. Furthermore, direct post-training of these large models on medical data faces two hurdles: first, the scarcity of high-quality, aligned image–text pairs in this niche field limits effective feature alignment [15]; second, aggressive instruction tuning [16] carries the risk of catastrophic forgetting, where the model’s inherent reasoning and generalization capabilities are degraded in favor of rote memorization of the training set with formatted instructions [17,18,19].

Most critically, clinical decision-making is not merely a classification task; it must be evidence-based and transparent. Standard “black-box” end-to-end models (e.g., static tuned MLLMs) cannot dynamically interact with updated clinical guidelines. A reliable diagnostic system requires the visual acuity to identify the disease and the cognitive flexibility to retrieve and apply current medical standards.

To address aforementioned challenges, we propose HevaDx, an evidence-based agentic system bridging perception and reasoning, for the diagnosis and treatment recommendations of hemangiomas and vascular malformations. Our core insight is that visual diagnosis and clinical reasoning, while related, require different optimization strategies. Visual diagnosis relies on high-fidelity feature extraction [20], while treatment recommendation relies on logical deduction and knowledge retrieval. Therefore, rather than forcing a single MLLM to handle both, we design a cooperative agentic system. We employ a lightweight, visually-specialized model (DINOv2) [21] to extract subtle lesion features for precise diagnosis. This diagnostic output, combined with patient history, is then fed into an LLM (Qwen2.5-7B-Instruct) [22] equipped with Retrieval-Augmented Generation (RAG) [23,24,25]. This allows the LLM to leverage its superior reasoning capabilities to synthesize the diagnosis, patient history and retrieved clinical guidelines, ensuring recommendations are both accurate and clinically grounded. Experimental results demonstrate that HevaDx achieves a top-3 accuracy of 94.8% for diagnosis and 83.3% for treatment recommendations.

Our contributions are threefold:

- We construct a high-quality, expert-annotated cohort of 7565 VA cases to conduct a preliminary feasibility study of SOTA open-source MLLMs. This preparatory evaluation exposes the significant limitations of generalist models in specialized diagnostics.
- We introduce HevaDx, a novel modular system that explicitly decouples the clinical workflow into a visual specialist and a reasoning specialist. By combining a lightweight, adaptable visual encoder with a knowledge-augmented LLM, we achieve superior diagnostic accuracy.
- We establish a rigorous pipeline for dataset construction, incorporating strict quality control, Region of Interest (ROI) annotation, and class balancing strategies, mitigating the long-tail distribution problem inherent in clinical data. We also validate that a Retrieval-Augmented Generation (RAG) mechanism enhances clinical safety by transforming opaque model outputs into transparent, evidence-based reasoning chains grounded in medical guidelines.

## 2. Materials and Methods

### 2.1. The Large-Scale VA Dataset and Evaluations on Advanced MLLMs

#### 2.1.1. Data Collection and Annotation

The dataset used in this study is independently curated by the Departments of Plastic Surgery and Laser Aesthetics at the Ninth People’s Hospital, Shanghai Jiao Tong University School of Medicine. All images were collected from patients with vascular anomalies who attended the outpatient clinic between January 2019 and August 2025. Clinical photographs were acquired using a Canon EOS 80D DSLR (Canon Inc., Tokyo, Japan) camera equipped with an EF 50 mm f/1.4 USM prime lens in a standardized photography studio under controlled lighting, ensuring high-resolution and consistent visualization of lesion areas. All samples were obtained retrospectively from routine clinical practice. Each case was confirmed by pathological examination or by senior clinicians in accordance with the Guidelines for Diagnosis and Treatment of Hemangiomas and Vascular Malformations (2024 Edition) [5]. Crucially, the treatment labels in this dataset represent retrospective guideline-based expert consensus, rather than raw historical clinical decisions, which might be confounded by non-medical factors (e.g., patient economic status or institutional equipment limitations). To ensure the ground truth reflects the optimal therapeutic strategy: (1) diagnostic labels and treatment recommendations were independently annotated by at least two senior plastic surgeons or dermatologists; (2) in instances where the two experts provided inconsistent diagnostic categories or treatment suggestions (e.g., differentiating between first-line and second-line therapies), a third senior expert adjudicated the case. This adjudication process guarantees that the final annotation represents the authoritative consensus compliant with the 2024 Guidelines. All data were anonymized prior to analysis, ensuring that no patient-identifiable information was retained.

#### 2.1.2. Quality Control and Dataset Statistics

After initial data collection, all images and corresponding patient information underwent a rigorous quality control process. Images with poor resolution, blurring, improper framing, or incorrect labeling were excluded from the dataset. Cases with incomplete clinical records or formatting inconsistencies were also removed to ensure that the final dataset maintained high integrity and reliability for downstream analysis. This quality check process was performed independently by five trained research staff members, and any discrepancies were resolved by a senior clinician.

**Figure 1:**
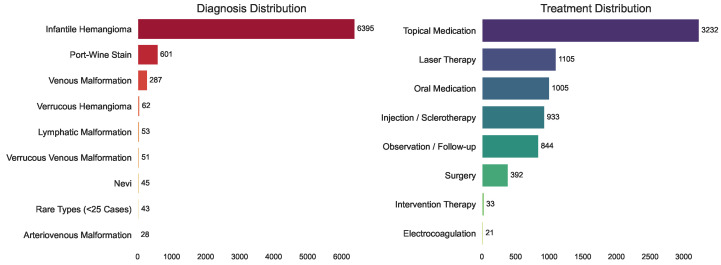
Diagnosis and treatment distributions. The dataset encompasses samples from 7565 patients, covering 14 VA subtypes and 8 treatment options, exhibiting a clinically typical long-tailed distribution.

Following quality control, the final dataset comprised a total of 7565 patients with various vascular anomalies. The distributions of diagnosis and treatment options are summarized in Figure 1. The most common diagnosis was infantile hemangioma (6395 cases, 84.5%), followed by port-wine stain (601 cases, 7.9%), venous malformation (287 cases, 3.8%), and other low-frequency categories (combined as 282 cases, 3.7%) (low-frequency categories (less than 25 cases) were grouped into an “Rare Types” category). Top treatment options included topical medication (3232 cases, 42.7%), laser therapy (1105 cases, 14.6%), oral medication (1005 cases, 13.3%), and injection/sclerotherapy (933 cases, 12.3%). Surgical interventions, interventional therapies, electrocoagulation, and observation/follow-up accounted for the remaining cases. Additionally, females (5213, 68.9%) outnumbers males (2352, 31.1%) in our dataset.

By applying this systematic processing and annotation workflow, we ensured the accuracy, completeness, and reliability of the dataset for subsequent benchmarking and method development. Table 1 presents the complete summary of the dataset statistics.

#### 2.1.3. Evaluations on Advanced Open-Source MLLMs

The diagnosis of VA relies on the precise interpretation of fine-grained visual cues—such as color depth, texture patterns, and boundary morphology—features that are often underrepresented in the datasets used to train generalist MLLMs. To empirically identify the limitations of existing architectures and justify the necessity of a specialized system, we conducted a preliminary evaluation on advanced open-source MLLMs. We randomly sampled 480 cases from our VA dataset for evaluation, ranging from common hemangiomas to rare vascular malformations and simulating the challenging long-tail distribution encountered in real-world clinical practice. We restricted the parameter space to the 4B–32B range, facilitating practical clinical deployment (the selected range and open-source nature enable local deployment on standard enterprise-grade hardware (e.g., single NVIDIA A100 or RTX 4090), facilitating data privacy compliance within hospital intranets). We evaluate two categories of model architectures: (1) general-purpose models including Qwen2.5-VL series [26], Kimi-VL-16B [27] and LLaVA-v1.5-7B [28]; (2) medical-specialized models like MedGemma series [29] and LLaVA-Med-v1.5-Mistral-7B [30]. We report the top-1 and top-3 accuracy for both diagnosis and treatment recommendations.

The results (see Table 2) reveal two critical bottlenecks. First, even the top-performing generalist model (Qwen2.5-VL-32B) achieved a diagnostic accuracy of only 58.1%, outperforming medically pre-trained models like MedGemma-27B (35.6%). This suggests that general medical tuning does not inherently generalize to the specialized morphology of VAs, where standard visual encoders fail to resolve fine-grained diagnostic details. Second, a catastrophic capability collapse occurs when shifting to therapeutic planning, with the best treatment recommendation accuracy plummeting to 16.3%. This decline underscores a fundamental “reasoning gap”: the existing end-to-end models struggle to translate visual perceptions into evidence-based clinical logic. These findings empirically validate the necessity of a specialized system.

### 2.2. The HevaDx  System

**Figure 2:**
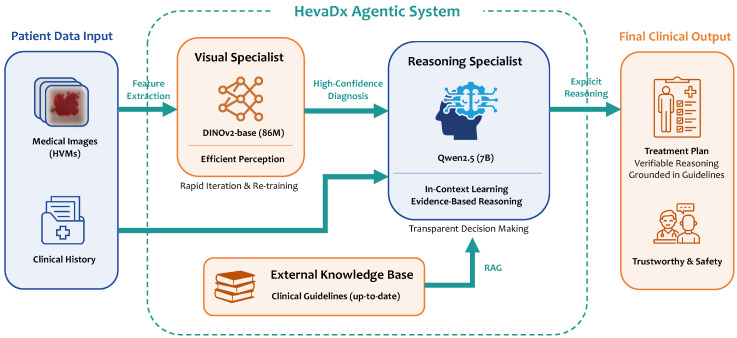
Overview of the HevaDx agentic system. HevaDx decouples the clinical workflow into two specialized components: a lightweight visual specialist for precise disease identification and a knowledge-augmented reasoning specialist for evidence-based treatment planning.

To address the limitations identified in the previous evaluation—specifically the trade-off between visual precision and reasoning capability—we propose HevaDx, a modular agentic system, as shown in Figure 2. Unlike traditional end-to-end architectures that attempt to optimize a single network for both perception and logic, HevaDx decouples the clinical workflow into two specialized components: a lightweight visual specialist for precise disease identification and a knowledge-augmented reasoning specialist for evidence-based treatment planning.

#### 2.2.1. The Visual Specialist: Efficient Perception with DINOv2

The diagnosis of vascular anomalies hinges on the detection of subtle morphological cues, such as the depth of red discoloration, the texture of the lesion surface, and boundary distinctness. MLLMs often struggle with these details due to the domain gap [31] between natural and medical imagery. To overcome this, we employ DINOv2 [21], a self-supervised vision transformer, as our dedicated visual backbone.

We selected DINOv2-base to harness the powerful visual feature extraction capabilities it obtained from massive pre-training. By fine-tuning this lightweight model with our VA cases, we ensure the model captures the fine-grained pathological visual features. Furthermore, the lightweight nature of the model (86M parameters) offers a critical practical advantage: adaptivity. As clinical data accumulation is a continuous process, medical AI systems require frequent updates. Retraining a massive MLLM is computationally expensive, whereas our decoupled visual specialist can be rapidly iterated and re-trained as new patient data becomes available, ensuring the diagnostic module remains current with minimal computational cost.

#### 2.2.2. The Reasoning Specialist: Transparent, Evidence-Based Decision Making

Once a high-confidence diagnosis is established by the visual specialist, the focus shifts to therapeutic management—a task requiring logical deduction rather than visual pattern recognition. We utilize an LLM (Qwen2.5-7B-Instruct, in which the reasoning specialist receives textual diagnostic results from the visual specialist) [22] as our reasoning specialist.

Our approach diverges from standard methods by strictly avoiding instruction-tuning on the LLM. Aggressive fine-tuning on limited medical data often degrades a model’s general reasoning capabilities (catastrophic forgetting). Instead, we leverage the model’s inherent in-context learning (ICL) capabilities [32,33,34]. The system operates by feeding the diagnostic output from the visual specialist, along with the patient’s clinical history, into the LLM. Crucially, we augment this input with relevant, up-to-date clinical guidelines retrieved from an external knowledge base. This design ensures two key clinical requirements: evidence-based reasoning and transparency. Specifically, by grounding the generation process in retrieved guidelines, the system minimizes hallucinations [35,36] and ensures recommendations align with current medical standards. Furthermore, unlike “black-box” end-to-end models, HevaDx produces explicit reasoning chains. Clinicians can verify exactly how a reasoning trajectory reach a specific treatment recommendation, fostering trust and safety in the clinical decision-making process.

## 3. Data Preprocessing and Setup

### 3.1. Dataset Stratification and Balancing

To prevent model bias toward high-prevalence diseases and ensure robust evaluation across the spectrum of vascular anomalies, we implemented a strict data balancing strategy. From our full dataset, we selected six representative disease categories with sufficient sample sizes: port-wine stain (PWS), infantile hemangioma (IH), venous malformation (VM), verrucous hemangioma (VH), verrucous venous malformation (VVM), and nevi.

We employed stratified sampling to construct an independent test set that reflects the diversity of the disease spectrum. Specifically, we randomly selected a fixed number of cases for each category, resulting in a total of 96 test samples: PWS (n=17), IH (n=17), VM (n=18), VH (n=12), VVM (n=15), and nevi (n=17). The remaining images constituted the training set (see Appendix B for more details). To address the long-tail distribution inherent in medical data, we applied a class balancing strategy during training set construction. For common disease categories exceeding 200 samples, we performed Random Undersampling to cap the count at 200. For minority classes with fewer than 200 samples, we retained all available high-quality images and applied Random Oversampling (duplication) to approximate a balanced distribution.

### 3.2. Data Preprocessing

Prior to training, we performed rigorous data cleaning and fine-grained annotation to maximize signal-to-noise ratio.

- Region of interest (ROI) annotation: We manually annotated bounding boxes for all lesion images. This step forces the model to focus its attention on the relevant pathological features, eliminating interference from background factors (e.g., clothing, medical equipment, or unrelated skin areas).
- Quality control: We conducted a secondary review to filter out low-quality samples. Images where the lesion location was ambiguous, or the diagnosis was clinically controversial, were excluded to prevent label noise.

Following this curation process, the final verified training set comprised the following distribution: PWS (n=200), IH (n=196), VM (n=200), VH (n=176), VVM (n=148), and nevi (n=175).

### 3.3. Implementation Details

- Visual Specialist Training: We trained the DINOv2-base model as our visual specialist. The training process was accelerated using a single NVIDIA A100 GPU (80 GB). We utilized the AdamW optimizer [37,38] with a learning rate of $5\times 10^{-5}$. The model was trained for 20 epochs with a batch size of 16.
- Reasoning Specialist Setup: For the reasoning component, we employed a Retrieval-Augmented Generation (RAG) framework. We constructed a specialized external knowledge base derived from the physician-summarized Guidelines for Diagnosis and Treatment of Hemangiomas and Vascular Malformations (2024 Edition) [5]. This ensures that the LLM’s (Qwen2.5-7B-Instruct) treatment recommendations are grounded in the latest clinical evidence. Detailed specifications regarding the chunking strategy, the rationale for choosing RAG, the knowledge update mechanism, and the prompt are provided in Appendix A.
- Metrics: To comprehensively assess system performance, we report the top-1 and top-3 accuracy for both the diagnosis task and the treatment recommendation task. Given that VA management often involves a hierarchy of valid therapeutic options (e.g., strictly observing a stable lesion is a valid alternative to laser therapy), we interpret these metrics through a nuanced lens. Specifically, top-1 accuracy quantifies the system’s precise alignment with the primary consensus gold standard (the adjudicated “best” option). Complementing this, top-3 accuracy serves as the critical indicator of clinical admissibility and guideline consistency. This metric assesses whether the consensus gold standard is retained within the model’s high-probability candidates, which captures the system’s alignment with the broader therapeutic consensus, accommodating the flexibility of clinical decision-making beyond a rigid single-label prediction. The evaluation was conducted using the independent test set described in Section 3.1. Note that since the reasoning specialist need to receive diagnostic results from the visual specialist to make further actions, the top-1 and top-3 accuracy for treatment recommendations are both based on the top-1 diagnosis. Furthermore, to provide a robust measure of statistical uncertainty, we report the 95% confidence intervals [CIs] for all metrics, estimated via a non-parametric bootstrap procedure with 1000 iterations. we also report class-specific Recall, Precision, and F1-score.

## 4. Results

### 4.1. Main Results

The performance of the proposed HevaDx system, as summarized in Table 3, validates the effectiveness of the decoupled agentic architecture in addressing the clinical requirements of VA management. While the preliminary evaluation in Section 2.1.3 identified significant bottlenecks in general-purpose models (note that for the final system evaluation, the diagnostic scope was refined following the class-balancing and quality control protocols described in Section 3.1), HevaDx attained a top-1 diagnostic accuracy of 75.0% and a top-3 accuracy of 94.8%. These results suggest that a specialized visual backbone, such as DINOv2, is better suited to resolving the fine-grained visual ambiguities inherent in vascular anomalies than standard generalist encoders.

More importantly, the system successfully addressed the previously observed “reasoning gap” in therapeutic decision-making. By explicitly decoupling perception from reasoning and grounding the process in retrieved clinical guidelines, the system achieved a treatment top-1 accuracy of 62.5% and a top-3 accuracy of 83.3%. This performance gain supports the hypothesis that an evidence-based agentic framework provides a more stable foundation for complex clinical workflows than end-to-end MLLM approaches, particularly in specialized domains where precision and adherence to guidelines are paramount.

A detailed per-class evaluation (see Table 4) further elucidates the diagnostic robustness of HevaDx. The system achieved exceptional F1-scores in identifying IH (0.882), which are the most prevalent category in clinical practice. While some visual similarities between PWS and VM resulted in a slightly lower precision for PWS (0.583), the high recall (0.824) ensures that these cases are effectively captured for secondary expert review. Furthermore, despite the inherent morphological complexity and limited sample sizes of rarer types like VH, the system maintained balanced performance with F1-scores exceeding 0.63. This class-level consistency, as reflected by the Macro-F1 score of 0.744, confirms that the proposed architecture effectively mitigates the long-tail distribution challenge and provides reliable diagnostic support across the full spectrum of the evaluated vascular anomalies.

### 4.2. Ablation Study on Data Preprocessing

To quantify the impact of our rigorous data curation pipeline—specifically the region-of-interest (ROI) annotation and class balancing—we conducted a comparative analysis between a model trained on raw, noisy data and one trained on our refined dataset.

**Figure 3:**
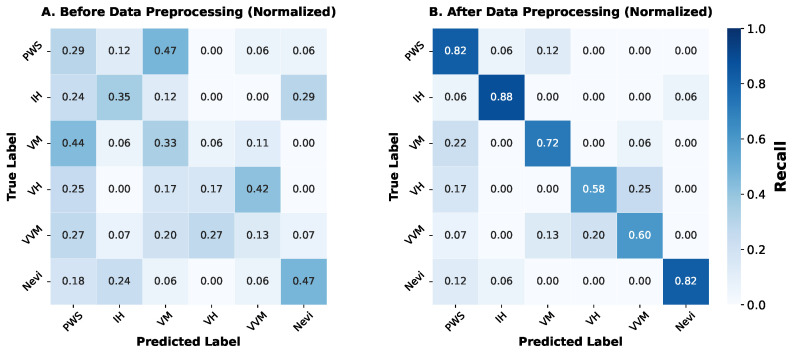
Resolution of clinical mimicry via data preprocessing. The model trained on preprocessed data demonstrates strong diagonal dominance, indicating robust correct classification.
- Enhancement of Discriminative Capability: The quantitative improvement is visualized in Figure 4. The model trained on preprocessed data exhibited dramatic performance gains across all disease categories. Notably, the F1-score for PWS surged from 0.39 to 0.88, and IH improved from 0.50 to 0.88. Even for morphologically complex subtypes like VH, which previously suffered from extremely low recognition (F1 = 0.21), the preprocessing strategy restored the model’s discriminative capability, raising the F1-score to 0.64.

- Resolution of Clinical Mimicry: The normalized confusion matrices further elucidate how preprocessing mitigates phenotypic confusion. As shown in Figure 3A (Before Preprocessing), the baseline model struggled with clinical mimicry, appearing unable to distinguish intrinsic lesion features from background noise. For instance, in the raw setting, PWS was frequently misclassified as VM (8 out of 17 cases), resulting in a recall of only 0.29. Similarly, VVM was heavily confused with PWS and VH, achieving a recall of just 0.13.
  In contrast, Figure 3B (after preprocessing) demonstrates strong diagonal dominance, indicating robust correct classification. The rigorous ROI annotation forced the visual encoder to attend to fine-grained texture and boundary features rather than background artifacts. Consequently, the confusion between PWS and VM was drastically reduced (only 2 misclassified), raising the PWS recall to 0.82. Although some confusion persists between the highly similar “verrucous” subtypes (VH and VVM), the overall class separability has been significantly enhanced, confirming that high-quality data curation is a prerequisite for resolving the long-tail distribution in vascular anomaly diagnosis.

### 4.3. Qualitative Analysis on Reasoning Specialist

To strictly isolate and evaluate the logical deduction capabilities of our reasoning specialist, we conducted a controlled experiment where the ground-truth diagnostic labels were directly provided to the reasoning specialist. This setup effectively bypasses visual perception errors, allowing us to assess HevaDx’s ability to map a confirmed diagnosis to an appropriate therapeutic regimen based on retrieved guidelines.

**Figure 5:**
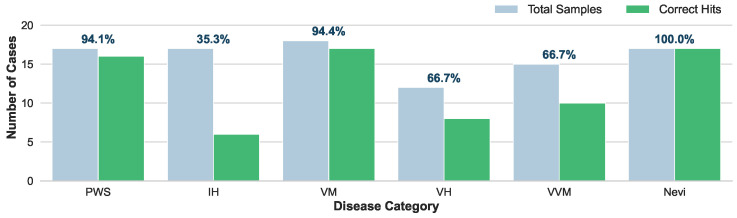
Performance analysis of the reasoning specialist on treatment recommendations. The reasoning specialist shows high efficacy in categories with highly standardized treatment protocols.

As illustrated in Figure 5, HevaDx demonstrates high efficacy in disease categories with highly standardized treatment protocols. For nevi (N=17), it achieved 100% accuracy (17/17 hits). Since surgical excision is the dominant gold standard for nevi, the model easily aligned with clinical consensus. For VM and PWS, HevaDx achieved 94.4% (17/18) and 94.1% (16/17) accuracy, respectively. Specifically, for PWS, the guideline recommendation is overwhelmingly “Laser Therapy”, and for VM, it is “Injection/Sclerotherapy”. The system’s high success rate here confirms its ability to correctly retrieve and apply strong evidence from the provided guidelines.

In contrast, IH represents a complex decision boundary, achieving a significantly lower top-1 accuracy of 35.3% (6/17). This discrepancy is not a failure of reasoning, but a reflection of clinical complexity. The ground truth for IH varies widely among “Oral Medication” (n=7), “Topical Medication” (n=3), “Surgery” (n=2), and “Observation/Follow-up” (n=3), often depending on subtle patient-specific factors (e.g., age, tumor depth, growth phase) that may be detailed in the medical history. The model displayed a distinct preference for “Oral Medication” (propranolol, predicting 14/17 cases), which is the first-line systemic therapy in current guidelines. While this lowered top-1 accuracy against a diverse ground truth, it reflects a safe and guideline-adherent baseline. Importantly, when expanding the evaluation to top-3 accuracy, the system achieved a remarkable 95.8% success rate across all categories. This indicates that even when the model’s primary recommendation differs from the specific clinical choice, the correct treatment is almost invariably captured within its top candidates.

To summarize, unlike “black-box” end-to-end models that might hallucinate treatments based on statistical correlations, our reasoning specialist grounds its decisions in explicit textual evidence. The high top-3 accuracy confirms that the system effectively narrows the search space to clinically valid options. For complex cases like IH, the system serves as a “safety net”, proposing the standard-of-care (e.g., oral medication) while allowing the clinician to refine the final choice based on specific patient nuance.

## 5. Discussion

Our study provides a critical reassessment of the application of MLLMs in specialized medical domains. The comparative evaluation in Section 2.1.3 revealed that current “generalist” state-of-the-art models, despite their massive parameter counts, struggle significantly with the fine-grained visual classification of VAs, achieving a diagnostic accuracy of only roughly 58%. More concerning was the “reasoning gap”, where treatment recommendation accuracy plummeted to 16.3% due to a lack of domain-specific grounding. In contrast, our proposed HevaDx system demonstrates that a modular, agentic architecture is superior for this task. By decoupling perception from reasoning, HevaDx achieved a top-3 diagnostic accuracy of 94.8% and a treatment accuracy of 83.3%. This validates our core hypothesis: specialized visual encoders are necessary to resolve clinical mimicry, while RAG is essential for bridging the gap between identifying a lesion and prescribing an evidence-based therapy.

Furthermore, our ablation studies underscore that high-quality data curation is as critical as model architecture. The dramatic improvement in F1-scores across all disease categories—particularly for morphologically complex subtypes like VH (improvement from 0.21 to 0.64)—confirms that rigorous ROI annotation and class balancing are prerequisites for handling long-tail medical distributions. Beyond accuracy, the qualitative analysis of the reasoning specialist highlights the system’s value as a transparent clinical assistant, while the model exhibited lower top-1 agreement in complex, multimodal treatment scenarios like IH (35.3%), its high top-3 accuracy and strict adherence to first-line guidelines (e.g., oral medication) indicate that it functions effectively as a safety net. Unlike opaque end-to-end models, HevaDx provides verifiable reasoning chains grounded in established guidelines, fostering the trust required for clinical collaboration.

Despite these promising results, several limitations must be acknowledged. First, regarding diagnostic nomenclature, our study retained the historical label “verrucous hemangioma” (VH) to align with the retrospective clinical records used for training. We explicitly acknowledge that VH is a misnomer; according to the latest ISSVA classification, this entity is biologically a verrucous venous malformation (VVM) characterized by somatic MAP3K3 mutations, rather than a vascular tumor. Future iterations will strictly adopt the updated VVM nomenclature to prevent conceptual ambiguity between malformations and tumors. Second, the current system covers only six major disease categories, omitting other critical entities such as arteriovenous malformations (AVM) and lymphatic malformations (LM). The exclusion of AVM was primarily due to the scarcity of confirmed cases in our dataset. Regarding LM, despite being a major category in the ISSVA classification, its exclusion was necessitated by three specific constraints: (1) Sample scarcity: The number of confirmed LM cases with high-quality surface imaging in our cohort was insufficient to support deep learning training. (2) Annotation ambiguity: Unlike hemangiomas or capillary malformations which present distinct cutaneous boundaries, LMs often manifest as diffuse, subcutaneous swellings with indistinct margins, making the precise annotation of Regions of Interest (ROIs) highly subjective and inconsistent. (3) Diagnostic modality mismatch: The definitive diagnosis of LM—particularly deep-seated or macrocystic types—predominantly relies on cross-sectional imaging (Ultrasound, MRI) and diagnostic puncture rather than surface photography alone. Consequently, including LM would have introduced significant label noise into a vision-based diagnostic framework primarily designed for cutaneous vascular anomalies. Third, while HevaDx has significantly improved diagnostic accuracy compared to baseline AI models, it still lags behind the nuanced reasoning of experienced board-certified clinicians, particularly in handling edge cases. Finally, our experiments were conducted retrospectively; prospective testing in a real-world clinical setting is required to validate the system’s efficacy and to fully address the ethical implications of AI-assisted diagnosis. To make the system comprehensively useful, future work must focus on continuous data collection and the integration of multi-modal data (e.g., MRI) to encompass the full spectrum of vascular anomalies.

## 6. Conclusions

In this study, we addressed the gap between general-purpose AI capabilities and the specialized requirements of diagnosing VAs. Our comprehensive evaluation revealed that while large foundation models possess strong general reasoning, they falter in the specific tasks of distinguishing VA subtypes and formulating safety-critical treatment plans. We proposed HevaDx, a novel agentic system that decouples perception and reasoning to overcome these bottlenecks. By combining a dedicated visual encoder with a guideline-retrieving LLM, our system achieves high performance while ensuring the transparency and interpretability essential for clinical adoption. Our experimental results show that a rigorous pipeline for dataset construction and data cleaning is essential for medical diagnostic tasks. Lastly, HevaDx shows that a modular, evidence-based approach is superior to “black-box” end-to-end paradigms for complex medical decision-making, paving the way for trustworthy medical AI assistants.

## Figures and Tables

**Figure 4:**
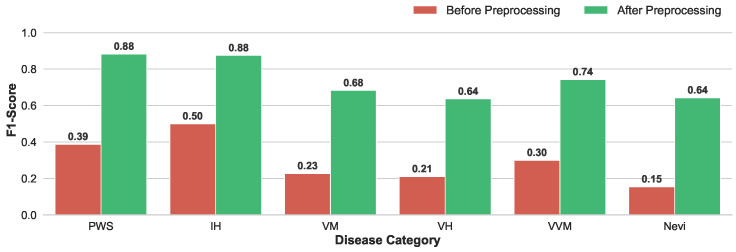
Enhancement of discriminative capability via data preprocessing. Through data preprocessing, the model’s ability to distinguish various diseases is enhanced, as evidenced by a marked increase in the F1-score.

**Table 1:** Statistical summary of the dataset.

Characteristic	Value/Details
Patients	7565 Cases
Disease	14 Types
Treatment	8 Options
Gender	Male & Female
Age	0–70 (Years)
Lesions	154 Body Sites

**Table 2:** Comprehensive evaluations on diverse open-source models (%). We report the top-1 and top-3 accuracy for both diagnosis (DiagAcc) and treatment recommendations (TreatAcc). Advanced MLLMs perform poorly on the task of diagnosis and treatment recommendations for VA.

Model	DiagAcc@1	DiagAcc@3	TreatAcc@1	TreatAcc@3
General-purpose models				
Qwen2.5-VL-7B-Instruct	48.8	61.9	10.6	30.6
Qwen2.5-VL-32B-Instruct	58.1	67.5	16.3	35.6
LLaVA-v1.5-7B	14.4	40.6	8.8	15.6
Kimi-VL-16B	43.1	62.5	15.6	38.1
Medical-specialized models				
MedGemma-4B	6.3	26.9	11.9	26.9
MedGemma-27B	35.6	66.9	13.1	36.9
LLaVA-Med-v1.5-Mistral-7B	22.5	53.1	10.6	28.1

**Table 3:** Main experimental results of the proposed HevaDx agentic system. Performance is reported as % [95% CI] calculated via 1000 bootstrap iterations. HevaDx shows high reliability across both diagnostic and therapeutic decision-making tasks.

Method	DiagAcc@1	DiagAcc@3	TreatAcc@1	TreatAcc@3
HevaDx	75.0 [66.7–83.3]	94.8 [89.6–99.0]	62.5 [53.1–71.9]	83.3 [75.0–90.6]

**Table 4:** Detailed class-specific performance metrics for diagnostic classification (Top-1). Precision, Recall, and F1-score are calculated for each of the six VA categories.

Category	Precision	Recall	F1-Score
PWS	0.583	0.824	0.683
IH	0.882	0.882	0.882
VM	0.765	0.722	0.743
VH	0.700	0.583	0.636
VVM	0.692	0.600	0.643
nevi	0.933	0.824	0.875
Macro Average	0.759	0.739	0.744 [0.644–0.828]

**Figure A1:**
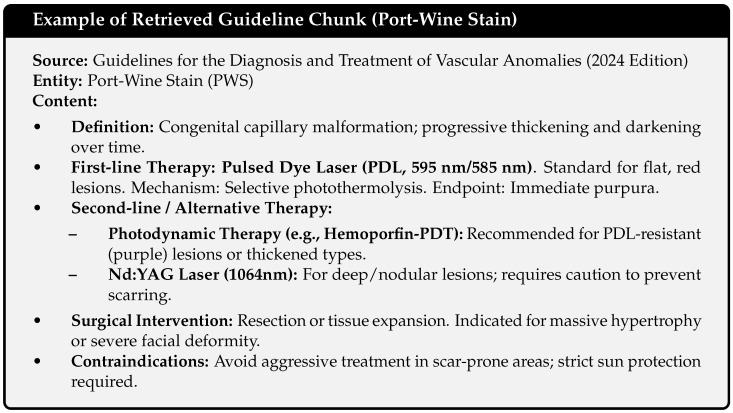
A representative example of a structured retrieval chunk for Port-Wine Stain (PWS). The content is extracted and summarized from the 2024 Guidelines to facilitate precise logic grounding for the reasoning specialist.

**Figure A2:**
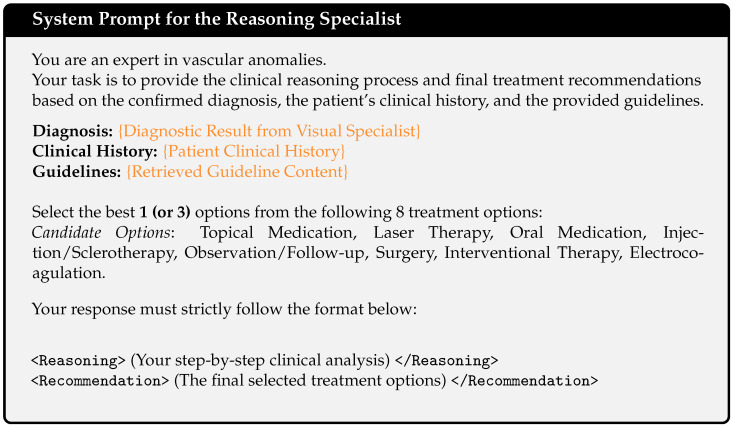
The specific prompt template designed for the reasoning specialist. By incorporating the visual diagnosis, patient history, and retrieved guidelines, the system is instructed to generate structured reasoning and treatment recommendations.

## Data Availability

The data presented in this study are not publicly available due to patient privacy and ethical restrictions consistent with the approval from the Ethics Committee of Shanghai Ninth People’s Hospital.

## Abbreviations

    The following abbreviations are used in this manuscript:

VA	Vascular Anomaly
PWS	Port-wine stain
IH	Infantile Hemangioma
VM	Venous Malformation
VH	Verrucous Hemangioma
VVM	Verrucous Venous Malformation
LLM	Large Language Model
MLLM	Multimodal Large Language Model
RAG	Retrieval-Augmented Generation
SOTA	State-of-the-art
ICL	In-Context Learning
ROI	Region of Interest

## Appendix A. Technical Implementation of the Reasoning Specialist

To ensure the treatment recommendations generated by HevaDx are both accurate and clinically verifiable, we implemented a specialized RAG framework grounded in the Guidelines for Diagnosis and Treatment of Hemangiomas and Vascular Malformations (2024 Edition) [5]. This section details the data construction, architectural rationale, and maintenance protocols. We also provide the prompt template used by the reasoning specialist (see Figure A2).

### Appendix A.1. Disease-Centric Chunking

Standard fixed-size chunking strategies (e.g., sliding windows of 512 tokens) often sever the logical flow of clinical protocols, leading to fragmented or decontextualized retrieval. To address this, we employed a Disease-Centric Chunking strategy.

Specifically, the knowledge base was constructed through a rigorous process where senior physicians manually extracted and synthesized core content directly from the Guidelines for Diagnosis and Treatment of Hemangiomas and Vascular Malformations (2024 Edition) [5]. Each chunk represents a physician-summarized therapeutic module for a specific VA subtype (e.g., port-wine stain, PWS), strictly adhering to a standardized schema: Definition → Diagnosis → First-line Therapy → Second-line/Alternative Therapy → Surgical Intervention → Contraindications.

For retrieval, we utilized a dense retriever based on the BGE-M3 architecture. This model encodes a composite query—synthesized from both the visual specialist’s diagnostic output and the patient’s textual clinical history—into high-dimensional vectors. To strictly anchor the retrieval trajectory to the identified disease category and prevent semantic drift caused by variable-length clinical histories, the diagnostic term (e.g., “port-wine stain”) is replicated 25 times within the query string. This heuristic weighting ensures that the retrieved guidelines are primarily determined by the diagnosis, while patient history serves as secondary context for refinement. As illustrated in Figure A1, the retrieved passage for PWS is not a raw text segment but a structured summary that explicitly prioritizes Pulsed Dye Laser (PDL) as the gold standard while presenting Photodynamic Therapy (PDT) and surgery as conditional alternatives for hypertrophic or resistant lesions. This structural integrity ensures the reasoning specialist receives hierarchical clinical knowledge rather than disjointed facts.

### Appendix A.2. Rationale for RAG over Rule-Based Systems

While a deterministic rule-based system (e.g., a lookup table mapping diagnostic labels to specific chunks) is technically feasible for the current closed-set evaluation of six diseases, we selected a RAG architecture to ensure the system’s long-term extensibility and robustness. This decision is driven by two critical architectural advantages:

- Decoupling Inference from Knowledge Management: A rule-based approach necessitates maintaining a synchronized code module that explicitly maps diagnostic outputs to document indices. As the system may expand to cover more subtypes in the ISSVA classification, this hard-coded logic would introduce significant deployment complexity and maintenance overhead. RAG effectively decouples the inference engine from the knowledge repository. This allows for the seamless expansion of disease categories by simply ingesting new vectorized chunks into the database, without requiring any modifications to the underlying deployment code or inference logic.
- Adaptability to Heterogeneous Knowledge Sources: Currently, the knowledge base is organized around disease-centric chunks. However, future iterations of the system could incorporate heterogeneous information sources that are not strictly disease-specific, such as symptom-management protocols, cross-disease contraindications, and unstructured expert clinical notes. Rule-based systems, which rely on rigid key-value pairing (i.e., Diagnosis → Guideline), fail to retrieve relevant context when the information is not taxonomically indexed by a specific disease name. In contrast, the semantic retrieval mechanism of RAG is agnostic to the data structure, allowing it to flexibly retrieve and synthesize context from diverse sources based on semantic relevance rather than rigid rules.

### Appendix A.3. Knowledge Update Mechanism

Clinical guidelines for VA could be updated periodically (e.g., the transition from the 2019 to the 2024 edition). When new guidelines are released, the outdated vector chunks are removed and replaced with newly vectorized summaries in the vector database. By leveraging the in-context learning (ICL) capability of the underlying LLM, the reasoning specialist can dynamically adapt to these retrieved updates, ensuring immediate alignment with the latest evidence-based protocols without requiring computationally expensive model retraining or fine-tuning.

## Appendix B. Justification of Test Set Size and Statistical Robustness

### Appendix B.1. Data Distribution Constraints

As detailed in Figure 1, the raw dataset exhibits a severe long-tail distribution. IH accounts for over 80% of the total cases, whereas other critical subtypes like VM and VVM are extremely rare, with only 40–70 confirmed cases available in total. To develop a clinically versatile system capable of identifying these rare but distinct entities, we filtered the dataset to include only classes with at least 40 confirmed samples. This resulted in the six target categories. For the rare classes (e.g., VM, VVM), allocating a rich split for testing would have left fewer samples for training, which is insufficient for learning robust visual features in a deep learning context. It is worth noting that LM, despite being a major category in the ISSVA classification, were excluded from this study due to three specific constraints. First, regarding sample scarcity, the number of confirmed LM cases with high-quality surface imaging in our cohort was insufficient to support the training of a deep learning model. Second, annotation ambiguity poses a significant challenge; unlike hemangiomas or capillary malformations which present distinct cutaneous boundaries, LMs often manifest as diffuse, subcutaneous swellings with indistinct margins, making the precise annotation of ROI highly subjective and inconsistent. Moreover, a fundamental diagnostic modality mismatch exists, as the definitive diagnosis of LM—particularly deep-seated types—predominantly relies on cross-sectional imaging (Ultrasound, MRI) and diagnostic puncture rather than surface photography alone. Consequently, including LM would have introduced significant label noise into a vision-based diagnostic framework primarily designed for cutaneous VAs.

To balance the trade-off between training efficacy and evaluation reliability, we adopted a stratified sampling strategy: we randomly selected approximately 16 independent samples from each of the six classes to form a balanced test set (n=96). This ensures that the evaluation metrics reflect the model’s performance across all disease types equally, rather than being dominated by the majority classes. The remaining samples were used for training. To mitigate the imbalance, we applied oversampling to the minority classes and undersampling to the majority classes, resulting in a balanced training set of 1095 images. This yields a training-to-test ratio of approximately 11:1, which prioritizes maximizing the exposure of the model to the limited available examples of rare diseases.

### Appendix B.2. Statistical Uncertainty Quantification

A small test set may introduce high variance in point estimates. To address this and prevent over-interpretation of the performance gains, we employed non-parametric bootstrapping to estimate the statistical uncertainty. Specifically, we generated 1000 bootstrap resamples from the test set and calculated the 95% Confidence Intervals (CIs) for the reported metrics. As shown in Table 3 and Table 4, the reported 95% CIs further substantiate the effectiveness and robustness of the proposed HevaDx.
